# The magnitude and associated factors of postpartum hemorrhage among mothers who delivered at Debre Tabor general hospital 2018

**DOI:** 10.1186/s13104-019-4646-9

**Published:** 2019-09-23

**Authors:** Daniel Habitamu, Yitayal Ayalew Goshu, Likenaw Bewuket Zeleke

**Affiliations:** 1 Department of Midwifery, Jimma Health Center, Jimma, Ethiopia; 2 Department of Midwifery, College of Health Sciences, Debre Tabor University, Debre Tabor, Ethiopia; 3 Department of Midwifery, College of Health Sciences, Debre Markos University, Debre Markos, Ethiopia

**Keywords:** Magnitude, Associated factors, Postpartum hemorrhage

## Abstract

**Objective:**

Since data related to postpartum hemorrhage in Ethiopia is scarce, this study was aimed to assess the magnitude and associated factors of postpartum hemorrhage among mothers who delivered in Debre Tabor general hospital.

**Results:**

In this study, one hundred forty-four mothers’ charts were reviewed which made the response rate 100%. This study revealed that the magnitude of postpartum hemorrhage was 7.6% (CI 6.2, 9.8). Chi-square test revealed that there was an association between postpartum hemorrhage and gravidity, parity, having antenatal care visit, and the previous history postpartum hemorrhage. This finding confirmed that uterine atony, retained placenta, and genital tract trauma were the most common leading cause of postpartum hemorrhage.

## Introduction

According to the World health organization (WHO) postpartum hemorrhage (PPH) defined as the blood loss of more than 500 milliliters following a vaginal delivery or more than 1000 ml following caesarian section [1, 2]. PPH also can be defined as any amount of vaginal bleeding following delivery that causes vital sign derangement or loss of 10% hemoglobin from the baseline [3, 4]. PPH can be caused primarily by atony uterus, retained tissue, genital tract tear, coagulation problem, and uterine rupture [5, 6].

Different studies reveal that mothers with postpartum hemorrhage exposed for a long period of hospital stay, blood transfusion, Sheehan’s syndrome, death, and other complications. PPH is one of the most critical problems in the public health system that imposes substantial financial costs on society [2, 4, 6–9].

According to WHO 2016 report, there are 216 million maternal deaths per 100,000 live births every year worldwide. 90% of maternal deaths happen in developing countries like Ethiopia [10]. WHO indicates that every year around 14 million mothers in the world suffer from vaginally bleeding following delivery. Based on world health organization data, probability of maternal mortality caused by postpartum hemorrhage is 1 in 1000 deliveries in developing countries, including Ethiopia. Moreover; all most all (99%) of maternal mortality due to vaginal bleeding following delivery occur in low- and middle-income countries [11].

In Ethiopia, reducing maternal mortality is the most challenging issue. Despite there is a decline in maternal mortality, the rate is as high as 412 per 100,000 live births [12]. World health organization reported that hemorrhage is the first leading case of maternal mortality globally [13]. According to two different studies which were done in Jima and Kersa revealed that postpartum hemorrhage was the first leading cause of maternal mortality which accounts for 54% and 46.5% maternal mortality respectively [14, 15].

The magnitude of PPH and its bad outcome is still a major health problem in developing countries including Subsaharan-African countries. The incidence of PPH in Pakistan, Uganda, and India was 6%, 7.4%, and 22.7% respectively [16–18]. In Ethiopia, the incidence of PPH vary from 1.4 to 9.69% [19–21].

The magnitude of postpartum hemorrhage is affected by age, gravidity, parity, ANC follow up, previous PPH, body max index, fetal macrosomia, mode of delivery, antepartum hemorrhage, duration of labor, and the number of the fetus [17, 20–23].

Despite maternal mortality in Ethiopia is too high, researches done in Ethiopia regarding PPH is extremely limited. Therefore this study was aimed to assess the magnitude and associated factors of postpartum hemorrhage among mothers who delivered in Debre Tabor general hospital. Findings from this study will be very important for policymakers and stakeholders in reducing maternal mortality. The result of this study also will be helpful for researches by serving as a piece of baseline information.

## Main text

### Methods

#### Study setting and design

This an institutional-based cross-sectional study was conducted in Debre Tabor general hospital from May 01 to 30, 2018. Debre Tabor general hospital is the only general hospital found in south Gondar zone and it is located 666 km away from Addis Ababa in the Northwest direction. Three health centers, three private clinics, and one general hospital are found in Debre Tabor town. This general hospital contains different departments like adult outpatient departments, emergency outpatient department, pediatrics ward, maternity ward, medical ward, surgical ward, laboratory units, pharmacy units, maternal and child health unit, neonatal intensive-care unit, and other units. According to south Gondar zone health department report, the hospital serves for a total of 2051, 738 populations who reside in south Gondar zone.

#### Study population

The study population of our study was all mothers who delivered at Debre Tabor general hospital from May 1, 2017, to April 30, 2018, the. Charts which were not complete were excluded from the study.

#### Sample size determination and sampling procedure

A single population proportion formula n = (Zα/2)^2^ P(1 − P)/d^2^ was used to calculate the sample size. Assumptions of 5.8% the population proportion of magnitude of PPH in Dessie referral hospital, 95% confidence interval, 4% of marginal error, and 10% non-response rate were used to calculate the required sample size. Finally, the required sample size was 144. The sampled mothers’ chart was selected using systematic random sampling.

#### Data collection

For data collection, a structured questionnaire was prepared after reviewing different literatures. Three diploma degree holder midwives were participated to collect the data. The data were collected using a pretested structured questionnaire via face to face interview. The questionnaire had two parts which were socio-demographic part and reproductive characteristics part.

The quality of the data was ensured through pretesting the questionnaire among 5% of sample size in Nefa Mewucha hospital and giving 2 days training regarding data collection process, the purpose of the study and ethical issues for data collectors and supervisors. In addition to this, the quality of data was ensured by supervising the overall data collection process.

#### Operational definition

- PPH: a woman would be considered developed PPH if the health care provider diagnosed and recorded on the chart.

#### Data management and analysis

The collected data were checked manually for completeness and consistency. After then, the data were coded, cleaned and entered to Epi data, software version-3.1. Finally, the data were analyzed using SPSS Version-20. Findings were summarized and presented using graphs and tables. To see the association between dependent and independent variables, the Chi-square test was computed. Variables which had a P-value < 0.05 in Chi-square test were considered as significantly associated with PPH.

#### Ethical considerations

Before data collection, the ethical clearance was obtained from Institute Review Board (IRB) of Debre Tabor University. This ethcical clearance was sought to Amhara Regional Health Bureau and support letter was obtained from Amhara Regional Health Bureau. The chief executive officer of the hospital was informed about the purpose of the study. Finally, the actual data collection was carried out after obtaining informed written consent from the chief executive officer of the hospital.

### Result

#### Socio-demographic characteristics

A total of 144 mothers’ chart were reviewed with a response rate of 100%. Less than half of mothers (43.1%) were found between 25 and 29 age groups. The mean age and standard division of the study participants were 20.7 and 7.75 respectively. Most of the mothers (87.5%) were married and all of the mothers (100%) were belongs Amhara ethnic group. Based on participants’ residency, the majority of mothers (70.1%) were from rural (Table 1).

**Table 1:** Socio-demographic characteristic of mothers in DTGH, Northwestern Ethiopia, 2018 (n = 144)

Characteristics	Frequency (N)	Percent (%)
Age		
15–19	13	9
20–24	38	26.4
25–29	62	43.1
30–34	17	11.8
≥ 35	14	9.7
Ethnicity		
Amhara	144	100
Residence		
Urban	43	29.9
Rural	101	70.1
Marital status		
Married	126	87.5
Divorced	13	9.3
Widowed	5	3.2

#### Obstetrics history

Among a total of 144 mothers, 11 (7.6%) of mothers diagnosed PPH (CI 6.2, 9.8). Among mothers whose chart reviewed (22.9%) were pregnant for the first time, 63.2% of them had 2–4 birth experience and only 13.9% of them had 5 or more birth. The majority (67.4%) of the women gave birth between gestational age of 37–42 and only 18.8% did not know the gestational age. Only 3 (2.1%) of the women had a previous history of PPH. Nine mothers (6.3%) had experienced previous placenta previa whereas five mothers (3.5%) developed antepartum hemorrhage during the current pregnancy (Table 2).

**Table 2:** Obstetrics characteristics of mothers in DTGH, Northwestern Ethiopia, 2018 (n = 144)

Variable	Category	Frequency	Percentage
Gravidity	1	33	22.9
	2–4	91	63.2
	≥ 5	20	13.9
Parity	1	46	31.9
	2–4	75	52.1
	≥ 5	23	16
Gestational age	< 37	12	8.3
	37–42	97	67.4
	> 42	8	5.6
	Unknown	27	18.8
History of previous C/S	Yes	9	6.3
	No	135	93.8
Previous PPH	Yes	3	2.1
	No	141	97.9
Current PPH	Yes	11	7.6
	No	133	92.4
Previous abruption placenta	Yes	6	4.2
	No	138	95.8
Previous placenta previa	Yes	9	6.3
	No	135	93.8
Current ante partum hemorrhage	Yes	5	3.5
	No	139	96.5
Delivery characteristics	Singleton	132	91.7
	Twin	12	8.3
Polyhydramnios	Yes	2	1.4
	No	142	98.6
Obstructed labor	Yes	7	4.9
	No	137	95.1
Prolonged labor	Yes	9	6.3
	No	135	93.8
Mode of delivery	Vaginal	123	85.4
	Caesarian section	21	14.6
Labor status	Spontaneous	129	89.6
	Induced	15	10.4
Labor augmented	Yes	18	14
	No	111	86
Third stage prolonged	Yes	5	3.5
	No	139	96.5
Method of placenta delivery	Spontaneous	2	40
	Manual placenta removal	3	60
Episiotomy	Yes	26	18.1
	No	118	81.9
Episiotomy extension	Yes	1	3.8
	No	25	96.2
Genital tract trauma other than episiotomy	Yes	2	1.4
	No	142	98.6
Uterine atone	Yes	3	2.1
	No	141	87.9
Retained placenta	Yes	6	4.2
	No	138	95.8

#### Associated factors

Since the logistic regression model didn’t fit, Chi-square test was computed to see the association between dependent and independent variables. Age (*χ*^2^ = 14.223, df = 4, P = 0.007), Gravidity (*χ*^2^ = 34.848, df = 2, P = 0. 000), Parity (*χ*^2^ = 29.231, df = 2, P = 0.000), ANC vsit (*χ*^2^ = 34.475, df = 1, P = 0.000), and previous PPH (*χ*^2^ = 38.442, df = 1, P = 0.00) were significantly associated with PPH (Table 3).

**Table 3:** Factors associated with PPH in DTGH, Northwestern Ethiopia, 2018 (n = 144)

Variable	Postpartum hemorrhage		χ^2^ (df)	P-value
	Yes	No		
Age				
15–19	1	13	14.223 (4)	0.007**
20–24	1	37		
25–29	2	59		
30–34	3	14		
≥ 35	4	10		
Residence				
Urban	3	40	0.038 (1)	0.845
Rural	8	93		
Marital status				
Single	1	6	4064 (3)	0.255
Married	8	116		
Divorced	2	9		
Widowed	1	2		
Gravidity				
1	1	33	34.848 (2)	0.00**
2–4	3 (27.27)	88		
≥ 5	7 (72.7)	12		
Parity				
1	1	46	29.231 (2)	0.00**
2–4	3	72		
≥ 5	7	15		
Gestational age				
< 37	0	12	3.649 (3)	0.302
37–42	7	90		
≥ 42	0	8		
Unknown	4	23		
ANC				
Yes	3	121	34.475 (1)	0.00**
No	8	12		
Abortion				
Yes	1	8	0.164 (1)	0.685
No	10	125		
Previous caesarian section				
Yes	4	17	4.536 (1)	0.033
No	7	116		
Previous PPH				
Yes	4	1	38.442 (1)	0.00**
No	7	132		

NB: ** indicates p-value < 0.05

### Discussion

In the current study, we assessed the magnitude and associated factors of postpartum hemorrhage among mothers who delivered at Debre Tabor general hospital. Assessing the magnitude and associated factors of postpartum hemorrhage is important for policymakers and stakeholders in reducing maternal mortality. This study revealed that the magnitude postpartum hemorrhage was 7.6% (CI 6.2, 9.8).

The result of the current study is similar to a study done in Uganda (7.4%) [17]. This finding also comparable with a study done in Bedele hospital (9.69%) [21]. The finding of this study is slightly higher than studies done in Pakistan (6%) [16]. The difference between two studies may be due to the sample size difference; the current study was carried out on small sample sizes whereas the previous Pakistan study was conducted on a large sample size. Another explanation may be due to the study setting difference and socio-demographic difference. This figure is also higher than studies done in Adis Ababa (1.4%) [19] and Dessie (5.8%) [20]. The difference figure between Addis Ababa study and the cure study maybe due to the low frequency of mothers who had antenatal visit in the current study. Many studies revealed that women who have antenatal visit are less likely to experience PPH. The possible reason for the difference between the current study and Dessie study is may be sample size, obstetrics characteristics, and socio-demographic difference. In the current study, most of the mothers were from rural and multipara. According to different studies, being from rural and multipara is at risk of developing PPH.

Based on the Chi-square test, age, gravidity, parity, previous PPH and ANC visit significantly associated with PPH. This study revealed that the proportion of PPH was high in women who had no ANC (72.72%) (*χ*^2^ = 34.475, df = 1, P = 0.000) follow up when compared with mothers who had ANC (27.3%) follows up. The findings of this study is in line with a study done in Dessie referral hospital [20]. Women who had ANC follow up may be screened early for risk factors of PPH and may get early treatment of the existing risk factors.

In our current study, we noted that gravidity (*χ*^2^ = 34.848, df = 2, P = 0. 000) and parity (*χ*^2^ = 29.231, df = 2, P = 0.000) were another predictor variable of PPH. This finding is similar to a study done in Bedele and Senegal [21, 22]. When parity and gravidity increase women’s myometrial muscular strength may get reduced due to the reduction of collagen fibers. Therefore; when gravidity and parity increases, the probability of developing PPH increases.

The finding of this study also indicated that previous history of PPH (*χ*^2^ = 38.442, df = 1, P = 0.00) was another variable which significantly associated with current PPH. This result is similar with a stud done in Uganda and Japan [16, 23] since PPH has a negative effect on muscular contraction; if a woman develops PPH once, she is at risk of developing PPH for next pregnancy.

This study concluded that the magnitude of PPH was relatively high in the study area. Gravidity, parity, ANC visit, and previous PPH were predictors of PPH. Reducing PPH by improving ANC followup is recommended.

## Limitation

The study might not be a true representative of the population since the study was hospital-based. This study also shares all the limitation of crosssectional study; the cause and effect of PPH might not be known. Since we used secondary data, there might be missed cases and variables. The direction and strength of association had not been known as it is one of the limitations of the Chi-square test.

## Data Availability

The datasets generated during the current study are available from the corresponding author based on reasonable request via email and phone call.

## Abbreviations

ANC: antenatal care
DTGH: Debre Tabor general hospital
PPH: postpartum hemorrhage
